# Causes and countermeasures for repeated outbreaks of hepatitis A among adults in Korea

**DOI:** 10.4178/epih.e2019038

**Published:** 2019-09-22

**Authors:** Moran Ki, Hyunjin Son, Bo Youl Choi

**Affiliations:** 1 Department of Cancer Control and Population Health, Graduate School of Cancer Science and Policy, National Cancer Center, Goyang, Korea; 2 Busan Center for Infectious Disease Control and Prevention, Pusan National University Hospital, Busan, Korea; 3 Department of Preventive Medicine, Hanyang University College of Medicine, Seoul, Korea

**Keywords:** Hepatitis A virus, Outbreak, Immunization, Catch-up, Epidemiology, Public health

## Abstract

The 2019 hepatitis A outbreak has become increasingly prevalent among adults in Korea and is the largest outbreak since that in 2009-2010. The incidence in the current outbreak is highest among adults aged 35-44 years, corresponding to the peak incidence among those aged 25-34 years 10 years ago. This may indicate a cohort effect in the corresponding age group. Causes of these repeated outbreaks of hepatitis A in Korea are low level of immunity among adults, Korean food culture that consumes raw seafood such as salted clam and inadequate public health system. Among countermeasures, along with general infectious disease control measures including control of the infectious agent, infection spread, and host, urgent actions are needed to review the vaccination policy and establish an adequate public health system.

## INTRODUCTION

This year (2019) has seen the appearance of a major outbreak of hepatitis A in Korea. In 2009-2010, a total of 22,886 hepatitis A patients was reported at the sentinel institutions [1,2] and health insurance reports indicated the treatment of 93,390 patients for hepatitis A as a primary disease. This year, 12,068 patients have been reported as of August 15 [3].

Hepatitis A virus (HAV) is a *Picornaviridae* RNA virus first isolated in 1979. The virus has been reported in some primates but humans are its main hosts [4].

While children have no or mild symptoms of cold, most adults have symptoms and can develop complications such as fulminant hepatitis, up to 80% of which can lead to death. Fortunately, an effective vaccine is available, which was first introduced as an optional vaccination in private medical institutions in Korea at the end of 1997. For people born in 2012 and after, a free vaccination program has been implemented in the country.

Thus, the generations of Koreans born in the 1970-1990s have benefited from improved environmental hygiene but have not participated in the free vaccination program. Therefore, the hepatitis A outbreak began when individuals born in the 1990s reached their 20s; as the age of this birth cohort increased, the hepatitis A incidence and severity have also increased. Therefore, the need for catch-up vaccination for this age group has been suggested [5].

This study examines the epidemiological characteristics of the 2019 hepatitis A outbreak and discusses the causes and countermeasures for repeated adult hepatitis A outbreaks in Korea.

### Ethics statement

This study received a review exemption from the Institutional Review Board of Hanyang University because it used published data (HYU-2019-04-021).

## EPIDEMIOLOGICAL CHARACTERISTICS

### Hepatitis A patients

**Figure 1.**
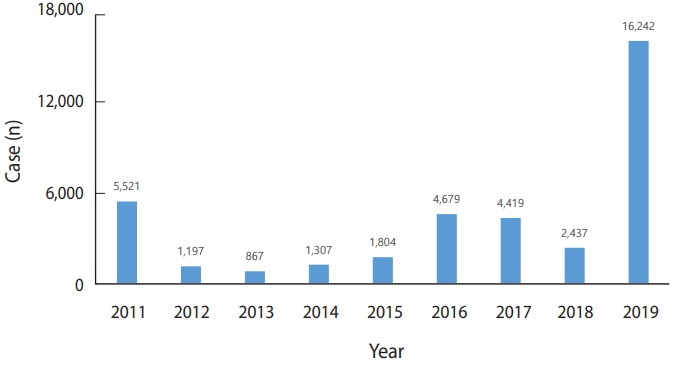
Reported cases of hepatitis A in Korea, 2011-2019 (as of October 13, 2019).

**Figure 2.**
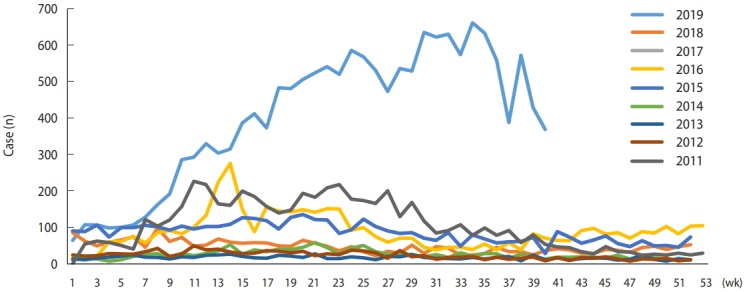
Weekly reported cases of hepatitis A in Korea, 2011-2019 (as of 40th week).

Hepatitis A was designated as notifiable infectious disease in 2011. Four to five thousand patients were reported each year in 2011, 2016, and 2017, with the lowest number of patients-867-reported in 2013. However, in 2019, the number of patients reported each week increased from the beginning of the year, resulting in 16,242 cumulative patient reports through October 13 of this year. As a result, this year’s outbreak will be the largest since hepatitis A was designated as notifiable disease (Figures 1 and 2) [3].

**Figure 3.**
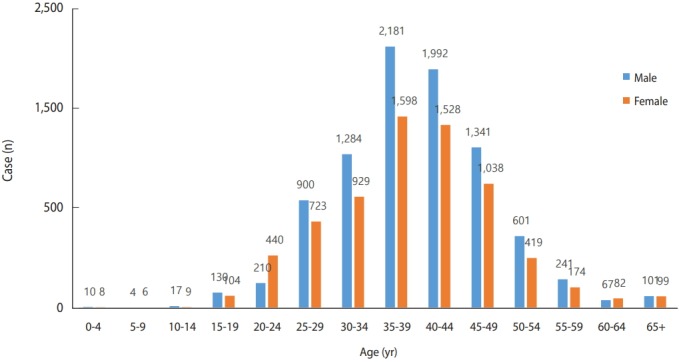
Age distribution of reported hepatitis A cases in Korea, 2019 (as of October 13, 2019).

The age of patients has gradually increased, with those aged 35-39 years showing the highest incidence in 2019, followed by those aged 40-44 years. This is exactly five years older than the age distribution of patients reported five years ago, in 2014. By sex, the incidence in male is higher than that in female, except for those aged 20-24 years, in which the incidence is higher in female. This is likely the effect of most male of this age serving in the military in Korea and hepatitis A vaccination during military service since 2015. In 2014, five years ago, 69 males and 63 females were reported in the 20-24-year age group, showing no significant difference, further supporting the proposed explanation for the difference in incidence in 2019 (Figure 3).

**Figure 4.**
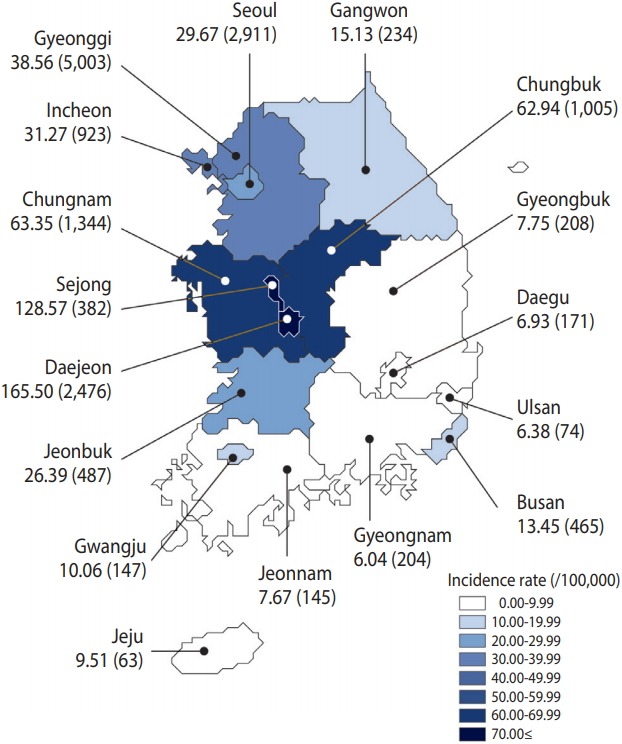
Incidence rate per 100,000 (n) of hepatitis A by area in Korea, 2019 (as of October 13, 2019).

As of October 13, 2019, the incidence rate was highest in Daejeon, followed by Sejong, Chungnam, Chungbuk, Gyeonggi, and Incheon (Figure 4).

### Hepatitis A deaths

**Figure 5.**
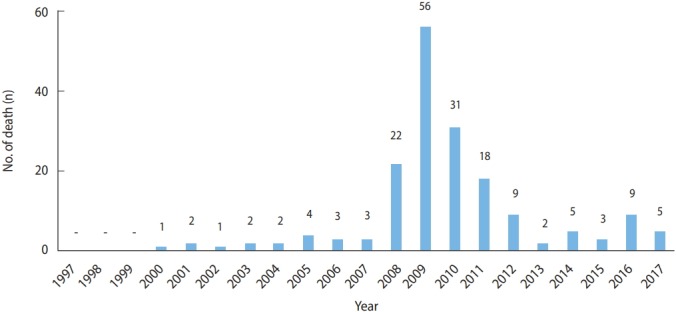
Hepatitis A deaths by year in Korea.

**Figure 6.**
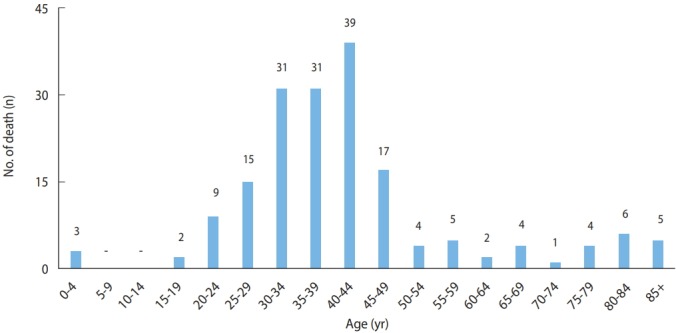
Age distribution of hepatitis A deaths in Korea, 2000-2017.

According to data from Statistics Korea, hepatitis A was the reported cause of death (B15) in 178 patients between 2000 and 2017. The highest annual number of deaths, 56, occurred in 2009, followed by 31 deaths in 2010. The 144 deaths in patients aged 20-49 years accounted for 81% of deaths due to hepatitis A. Among them, male accounted for 71% of deaths. Therefore, death due to hepatitis A primarily occurred among young male (Figures 5 and 6) (Supplemental Material 1).

### Hepatitis A virus seropositivity in Korea

According to the annual changes in immunoglobulin G (IgG) seropositivity rates, which indicate immunity against hepatitis A in Korea, the hepatitis A IgG seropositivity rate in group aged 0-9 years has been satisfactory at 70.0% since 2012 when the National Immunization Program (NIP) was introduced. The seropositivity of anti-HAV in individuals aged 10-19 years was 15.4% in 2005 and has increased steadily to 35.2% in 2014. Among those in their 20s, the seropositivity declined from 22.5% in 2005 to 8.7% in 2010, during the outbreak, and then rose again to 20.2% in 2014. The largest change in immunity levels was observed for individuals in their 30s; however, the seropositivity declined from around 69.6% in 2005 to 32.4% in 2014. Immunity levels also decreased in those in their 40s, from 97.9% to 79.3%, but the magnitude of the change was not as much as that for those in their 30s. Those over 50 years maintain high levels of immunity of over 98.0%. Thus, the 10-39-year age group has immunity levels of less than 40.0%, which corresponds to the birth cohorts in the 1970s and 1990s. As these cohorts age, the immunity level of Korean adults is likely to decrease [6,7]. According to the results of the 2015 Korea National Health and Nutrition Examination Survey, the seropositivity by age was 12.6% among participants in their 20s, 31.8% among those in their 30s, and 80.3% among those in their 40s, comparable to those in other reports (Table 1) [6,8].

## CAUSES OF THE LARGE OUTBREAK

The main mode of HAV transmission are person to person close contact and waterborne and foodborne transmission. The identified mode of hepatitis A infection in Korea are person to person, contaminated drinking water [9,10], and contaminated salted clams [11-14]. However, what is the reason that the scale of outbreak is increasing unlike in previous years? The most important causes can be summarized into four.

First, the immunity level of hepatitis A in adults is getting lower [15]. Until the 1960s birth cohorts in Korea, people acquired immunity from natural infections. However, for birth cohorts from the 1970s and later, the improved level of public hygiene led to reduced hepatitis A immunity. Hepatitis A vaccination was introduced in Korea in the late 1990s and the NIP was first implemented in the 2012 birth cohorts and later, thus increasing immunity levels in these birth cohorts. However, immunity levels in the earlier 1990s birth cohorts remain low. Thus, over time, the number of naturally infected adults born before the 1960s is decreasing and the proportion of adults without naturally-acquired immunity is increasing. In the context of the 2019 outbreak, adults aged 20-49 years had the highest levels of social activities, corresponding to birth cohorts from the 1970s to 1990s; i.e., the age group with low immunity levels.

Second is the change in Korean dietary culture. Chinese salted clams have been linked to the HAV in the current outbreak [11]. Salted clams are a traditional Korean food; however, recently, salted clams from China are imported and distributed more than domestic ones. These shellfish may contain high concentrations of HAV and were identified as a risk factor during the 2009 outbreak [16]. This seafood is salted without being cooked and HAV can survive in salted environments [17]. HAV is inactivated only when the salinity exceeds 10% [18]. In addition, the coastal areas of China, where the hepatitis A incidence is high, are more likely to be contaminated with the virus than in Korea. A 1988 hepatitis A outbreak in Shanghai affected approximately 300,000 patients, with an incidence among those who had eaten uncooked shellfish 22.9 times that in those who had not consumed the shellfish [19].

Third, the public health system in Korea is inadequate. Although Korea’s medical technology development is among the best in the world and the utilization of healthcare services and medical expenditures have increased significantly, the public health system has not correspondingly improved. Outbreaks of emerging infectious diseases are occurring worldwide and the threat of the importation of infectious disease and healthcare associated infection including antibiotic resistance is increasing. However, the public health system necessary to carry out effective countermeasures and the national budget allocated for public healthcare personnel and public health, in general, are inadequate. A typical consequence resulting from this vulnerability of Korea was the Middle East Respiratory Syndrome outbreak in 2015. Since then, the system and policies for recruiting epidemic intelligence officers and managing hospital infections have been introduced; however, the speed of the implementation is slow and fundamental issues such as the development of healthcare workers, healthcare delivery system, and consolidation of the public health organization system have not changed. The current hepatitis A outbreak was predicted from the 2009 outbreak. At that time, the low immunization rates of birth cohorts in the 1970s and 1990s were identified as the cause and catch-up vaccination was recommended as the most cost-effective measure to increase immunity level of the corresponding age groups [20,21]. The actual policy was to increase the 1-year-old vaccination rate to 90% by including hepatitis A vaccination into NIP, from the approximately 50% rate as an optional vaccination. These policies were the most inefficient in modeling research. As a result, as of 2019, immunity levels in children up to age 7 have increased significantly, but the lower immunity levels in older children and adults have not been addressed. At the end of 2010, hepatitis A was designated as a class 1 notifiable infectious disease, which includes waterborne and foodborne infectious diseases, due to the need for epidemiological investigations and systematic prevention and control. However, we should review and evaluate the reported improvement in epidemiological investigations and systematic prevention and control of hepatitis A over the last decade. Although the number of waterborne and foodborne infectious diseases outbreaks has been increasing annually in locations such as group catering centers, we have focused only on the management of respiratory infectious diseases and infrastructure establishment; thus, we should review whether we have neglected the epidemiological investigation, management, and prevention of waterborne and foodborne infectious diseases. As a matter of priority, an adequate system for epidemiological investigation and countermeasures against these infectious diseases should be in place, which will require substantial improvements in our capability to perform these actions at regional and basic regional government levels.

Fourth, the coordination of public health policy is also inadequate. For example, to cope with repeated hepatitis A outbreaks in the army, the Ministry of National Defense in Korea started single dose hepatitis A vaccination in 2013 for new recruits and expanded its scope to all recruits in 2015 [22]. As a result, hepatitis A outbreaks in the military have been greatly reduced. Unfortunately, only 70% of individuals develop immunity with the single-dose vaccination; therefore, a second vaccination is necessary. However, there is currently no system to link such information.

Therefore, there is a possibility of hepatitis A infection again after becoming a civil member of society following the end of military service. In addition, new recruits who have already received vaccination up to the second dose are unnecessarily re-vaccinated because there are no registered or linked data. Fortunately, this issue will be addressed by the vaccination records linkage agreement this year between the Korea Centers for Disease Control (KCDC) and the Ministry of National Defense. Children under 16 years (up to 18 years, depending on vaccine type) can receive vaccine for children that is half the cost, thus increasing group immunity of adolescents at an early age, and thus effectively contributing to the prevention of hepatitis A outbreaks [23]. Using adult vaccination in military recruits is wasteful in comparison.

Hepatitis A vaccination history should be managed from children to adults. The mandatory vaccination for children provided for free in private medical institutions under the NIP, the KCDC reports satisfactory levels of management, with a vaccination registration rate of 90% or higher in 2012. However, rotavirus or Bacillus Calmette–Guérin transdermal vaccines in children and most vaccines except for influenza and pneumococcal vaccines for adults aged 65 and older are not mandatory; thus, there is no management of vaccination registration.

Therefore, even if an adult has received hepatitis A vaccination at their own expense, they may have to undergo unnecessary tests or repeated vaccination if they cannot remember or lack an evidential record. As the number of adult vaccination items and the proportion of elderly individual increase, so does the importance of adult vaccination registration management.

## COUNTERMEASURES FOR HEPATITIS A OUTBREAKS

Three basic policies can be implemented to prevent infectious disease outbreaks. First, we should eliminate the infectious agents by identifying and isolating hepatitis A patients who are the source of viral spread. Early identification of hepatitis A patients allows prompt treatment and minimizes their contact with others. However, the identification and isolation of every hepatitis A patient is challenging since the initial symptoms are non-specific, similar to those of the common cold; moreover, some cases are asymptomatic but can still contribute to virus spread.

Second, we should block the spread of infection by removing or disinfecting contaminated water, food, toilet handles, and tools used in daily life, etc. to prevent contact by susceptible individuals. Thorough epidemiological surveys and investigations are needed to prevent further incidence by identifying contaminated foods or water and preventing their use or spread. It is also important to keep your hands clean in everyday life after using the bathroom. However, this method alone cannot completely block hepatitis A outbreaks.

Finally, the third method is to increase immunity among people at risk of infection so that they do not develop illness following exposure. Immunity can be acquired through infection at a young age (natural immunity) or vaccination (artificial immunity). Natural immunity is currently difficult to acquire in Korea and the recent implementation of mandatory infant vaccination under the NIP leaves the challenge of increasing immunity in adults [24]. However, it is practically difficult to administer the hepatitis A vaccine to all individuals. Therefore, all three policies should be deployed to prevent hepatitis A outbreaks. Which policy will be conducted as a priority will depend on the circumstances in each country. Immunization policies, in particular, depend on the hepatitis A incidence rate in each country. The World Health Organization recommends vaccination of individuals in countries with very low incidence of hepatitis A and rare outbreaks who are at high risk of hepatitis A infection (i.e., those travelling in high-prevalence regions, hemophiliacs, male homosexuals, those with illegal drug abuse, and jobs in contact with primates) [25]. In countries with a high incidence of hepatitis A, large-scale vaccination is not recommended as it will have little effect since many people tend to be infected when they are young and acquire natural immunity. However, large-scale vaccination is a cost-effective measure in countries, such as Korea, with large differences in immunity levels by age and region due to reduction in hepatitis A incidence [25]. The immunity levels are relatively low among Koreans born in the 1970s to 1990s and even lower in urban areas. In addition, the incidence, and complication and mortality rates of hepatitis A increase with age; thus, the hepatitis A fatality rate may increase in future outbreaks. Therefore, a large-scale catch-up vaccination policy is required for adults in their 20s to 40s. The hurdles to the introduction of such a policy, including securing budgets, vaccine supply, and low vaccination rates, can be overcome if with the establishment and implementation of a long-term plan of five years or more. As a short-term measure, organizations such as public health centers should provide adult vaccines at a lower cost than that of private hospitals to provide an alternative for those who are not vaccinated due to cost.

## CONCLUSION

The current hepatitis A outbreak in Korea has many implications. Developing countries with rapid economic growth leading to improved environmental hygiene may encounter unexpected consequences such as hepatitis A outbreaks. Timely infant vaccination should be introduced to improve herd immunity in certain age groups. Moreover, the introduction of an immunization registry system may be needed to provide adequate measures for effective control of hepatitis A.

Korea has a low level of immunity in the adult population and a high population density, with increasing numbers of people traveling abroad annually. Due to factors such as close contact in schools and workplaces, high healthcare services utilization, cultural habits of eating out with company, and shellfish aquafarming, Korea has higher risks of hepatitis A outbreaks compared to those in other countries. These characteristics also support the increased risk of outbreaks of new types of infectious diseases or healthcare-related infection. However, contrary to the high level of medical technology development in Korea and the high public expectations for health care, the public health systems fail to meet these expectations.

Temporary or one-off measures are ineffective against infectious disease outbreaks. Outbreak prevention requires early detection, prevention of further spread, and effective control to minimize damage. There is a pressing need for fundamental change and advances in the public health system in Korea.

## Figures and Tables

**Table 1.** Area and sex-adjusted seroprevalence (%) of anti-HAV from 2005 to 2014, in Korea^1^

	2005	2006	2007	2008	2009	2010	2011	2012	2013	2014	2015
Subjects (n)	1,140	1,642	2,050	6,207	14,101	60,846	83,586	76,906	84,102	93,665	-
Age (yr)											
0-9	33.4	52.7	42.4	50.7	69.8	65.8	53.9	71.0	65.4	67.7	-
10-19	15.4	19.0	20.0	25.5	23.2	19.0	26.4	33.8	35.4	35.2	42.1
20-29	22.5	29.2	19.1	17.2	11.9	8.7	13.3	16.2	16.3	20.2	12.6
30-39	69.6	67.6	63.8	58.6	48.4	40.7	37.3	34.3	32.1	32.4	31.8
40-49	97.9	96.7	94.7	91.4	89.1	87.9	86.1	84.1	80.8	79.3	80.3
50-59	98.7^2^	98.2^2^	98.0^2^	99.0^2^	98.8^2^	98.7	98.7	98.4	98.0	98.1	97.7
60+	-	-	-	-	-	99.2	98.1	99.3	99.5	99.6	-
Overall^3^	65.6	68.2	64.9	65.1	63.8	61.0	60.5	62.9	61.6	62.2	-

HAV, hepatitis A virus.

1 Seroprevalence of anti-HAV was adjusted by area from 2005 to 2009 and by area and sex from 2010 to 2014.

2 From 2005 to 2009, people over 50 years old presented in groups because of the small numbers and similar seroprevalence.

3 Overall seroprevalence was adjusted by age, area, and/or sex using the 2010 population.

Data sources from: 2005-2014: Kim et al. PLoS One 2017;12:e0170432 [6]; 2015: Lim et al. PLoS One 2017;12:e0189210 [8].

## SUPPLEMENTARY MATERIALS

Korean version is available at http://www.e-epih.org/.
