# Supplementary material for: Causes and countermeasures for repeated outbreaks of hepatitis A among adults in Korea
Source: Epidemiol Health. 2019 Sep 22;41:e2019038. doi: 10.4178/epih.e2019038 (PMC6883026; doi:10.4178/epih.e2019038)
Supplement: Supplementary file 1 [file epih-41-e2019038-supplementary.pdf]

Volume: 41, Article ID: e2019038

https://doi.org/10.4178/epih.e2019038

-SUPPLEMENTARY MATERIAL-

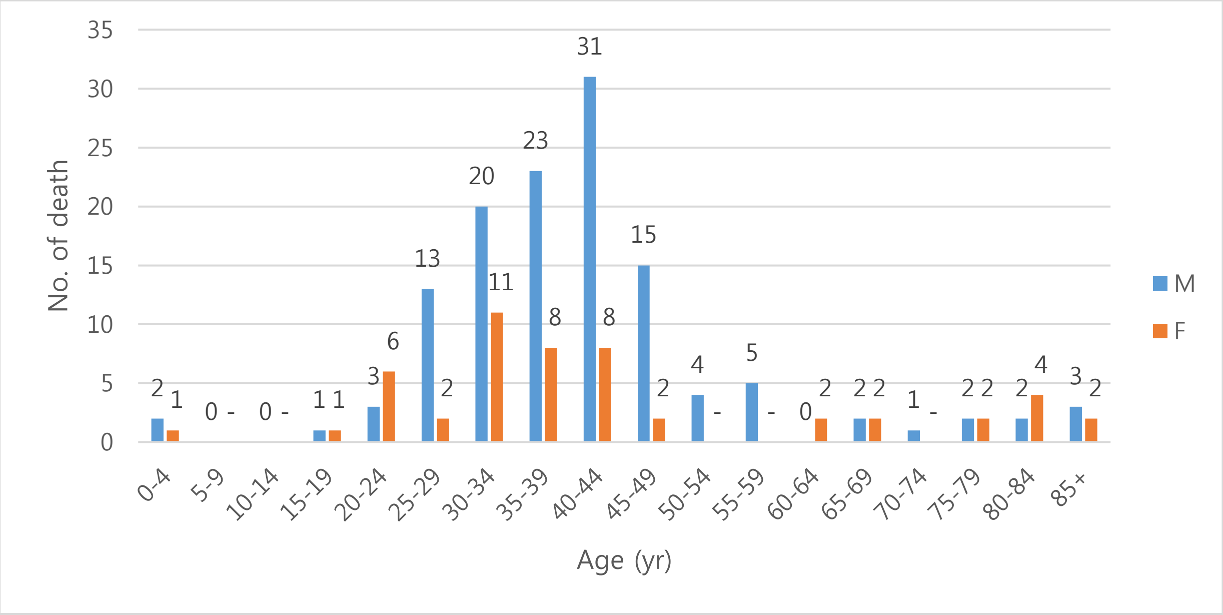

Supplementary Material 1. Age and sex distributions of hepatitis A deaths in South Korea, 2000–2017.
