# Supplementary material for: Causes and countermeasures for repeated outbreaks of hepatitis A among adults in Korea
Source: Epidemiol Health. 2019 Sep 22;41:e2019038. doi: 10.4178/epih.e2019038 (PMC6883026; doi:10.4178/epih.e2019038)
Supplement: Supplementary file 2 [file epih-41-e2019038-supplementary2.pdf]

Volume: 41, Article ID: e2019038

https://doi.org/10.4178/epih.e2019038

-Special Article-

Causes and countermeasures for repeated outbreaks of hepatitis A among adults in South Korea

(한국에서 반복되는 성인 A형 간염 유행의 원인과 대책)

Abstract

2019년 한국 성인에서 A형 간염 유행이 확대되는 양상이다. 10년전인 2009-2010년의 대규모 유행이후 가장 큰 규모의 유행이다. 이번에는 35-44세 발생 수준이 가장 높아서 10년전 25-34세 주 발생연령에서 정확히 10세가 증가한 양상이다. 이는 해당 연령 그룹의 코호트 효과라 할 수 있다. A형 간염의 반복 유행의 원인은 성인의 낮은 집단면역수준, 어패류 생식문화, 공중보건의료체계 미비 등으로 보인다. 이에 대한 대책은 일반적인 감염관리대책인 감염원관리, 전파관리, 숙주관리와 함께 효율적인 백신접종정책, 공고한 공중보건관리체계 마련 등의 근본적 대책마련이 시급하다.

Key words: Hepatitis A virus; Outbreak; Immunization, Catch-up; Epidemiology; Public health

Acknowledgements

본 연구는 대한민국 정부의 재원으로 방역연계범부처감염병연구개발사업(HI18C1903) 지원에 의하여 수행되었습니다(과제고유번호 HG18C0088).

Volume: 41, Article ID: e2019038

https://doi.org/10.4178/epih.e2019038

서론

2019년에 한국에서 A형 간염 대유행이 다시 발생하였다. 2009-2010년에 표본기관에서 신고된 환자는 22886명(1, 2), 건강보험에서 A형간염을 주상병으로 치료받은 환자는 93390명에 이르는 큰 유행이 발생하였고 이후 해마다 3천-5천명 정도의 발생을 보였으나 올해는 8월 15일까지 신고된 환자 수가 12,068 명에 이른다(3).

A형 간염 바이러스는 *Picornaviridae*에 속한 RNA 바이러스로 1979년에 처음 분리되었다. 몇몇 영장류에서도 발생하지만 주로 인간이 숙주이다(4).
소아에서는 증상이 없거나 감기증상 정도로 약한 증상을 보이지만 성인에서는 대부분 증상을 보이고 전격성 간염 같은 합병증도 나타날 수 있으며 이 경우에는 80%까지도 사망할 수 있다. 다행히 효과가 좋은 백신이 개발되어 있고 국내에서는 1997년말부터 선택접종으로 민간의료기관에서 사용하기 시작하였고, 2012년 출생자부터는 국가에서 무료접종을 시행하고 있다.
그렇다보니 우리나라의 1970-1990년대 출생자는 환경위생 개선의 혜택은 입었으나 예방접종의 혜택은 받지 못한 세대이다. 따라서 이 연령대가 20대에 이르는 1990년대부터 성인에서 A형 간염이 발생하기 시작하였고 이 출생코호트의 연령이 증가하면서 A형 간염 발생수준과 중증도도 증가하고 있다. 따라서 이 연령대를 대상으로 한 따라잡기 예방접종의 필요성이 제기되고 있다(5).

Volume: 41, Article ID: e2019038

https://doi.org/10.4178/epih.e2019038

이 글에서는 2019년도 A형 간염 유행의 역학적 특성을 살펴보고 우리나라에서 반복하여 발생하고 있는 성인 A형 간염 유행의 원인과 대책에 대하여 논의하고자 한다.

Ethics statement: This study received a review exemption from the Institutional Review Board of Hanyang University because it used published data (HYU-2019-04-021)

본론

역학적 특성

(A형 간염 환자 특성)

A형 간염 환자 신고는 2011년부터 전수 신고를 시행하고 있다. 2011, 2016년, 2017년에는 4천-5천명대의 환자가 신고되었고 신고 환자수가 가장 적었던 2013년에는 867명이 신고되었다. 그런데 2019년에는 연초부터 주별 신고 환자 수가 증가하여 10월 13일까지 누적 신고 환자수가 16,242 명에 이르렀고, 더욱이 주별 신고 환자수가 지속적인 증가세를 보이고 있어 우리나라에서 A형 간염 감시를 시작한 이래 가장 큰 유행을 겪고 있다(Fig.1, 2)(3).

환자들의 연령은 점차 높아져서 2019년에는 35-39세가 가장 높고 다음이 40-44세 이다. 이는 5년 전인 2014년 신고된 환자의 연령분포보다 정확히 5세가 증가한 것이다.

성별로는 남자가 여자보다 높은데 유독 20-24세 연령군에서는 여자가 남자보다 높게 나타나고 있다. 이는 이 연령대의 남자들이 군대에 있기도 하고, 2015년부터 군대에서 A형간염 예방접종을 시행한 효과라고 볼 수도 있다. 이는 5년 전인 2014년에는 20-24세 연령군에서 남자는 69명, 여자는 63명이 신고되어 남녀 차이가 없었던 것을 보아도 짐작할 수 있는 부분이다 (Fig.3).

Figure 1. Age distribution of reported hepatitis A cases in South Korea, 2019* (* as of August 15)

2019년 8월 15일까지 지역별 발생률을 보면 대전이 가장 높고 다음은 세종, 충북, 충남, 경기, 인천의 순서로 나타나고 있다. (Fig 4.)

(사망자 특성)

2000년부터 2017년까지 통계청 사망원인 자료에서 A형 간염이 사망원인(B15)으로 기록된 사람은 총 178명이다. 사망자가 가장 많았던 해는 2009년으로 56명이었고 그 다음이 2010년 31명이었다. 20-49세 연령의 사망자가 144명으로 전체 사망의 81%를 차지하였다. 또한 남성이 127명으로 71%를 차지하였다. 이는 A형 간염의 사망자가 주로 젊은 연령의 남성임을 의미한다 (Fig 5, 6).

(역학적 특성)

1. 국내 HAV 항체양성률

우리나라 a형 간염에 대한 면역을 보는 IgG항체 양성률의 연간 변화를 보면 0-9세에서는 A형 간염 백신 접종이 NIP에 포함된 2012년 이후에는 70%에 이르는 수준으로 양호하다. 10-19세는 2005년에는 15%수준이었으나 조금씩 올라서 2014년에는 35%로 나타났다. 20대는 2005년에 20% 수준에서 점차 감소하여 유행이 있었던 2010년에는 8.7%까지 감소하였다가 2014년에는 다시 20%수준으로 올랐다. 하지만 30대는 2005년 약 70%에서 2014년 32.4%까지 지속적으로 감소하여 가장 면역도 수준의 변화가 컸다. 40대도 면역수준이 감소하였으나 (97.9%에서 79.3%) 30대만큼 차이가 크지는 않았다. 50대 이상은 여전히 98%이상의 높은 면역수준을 유지하는 것으로 나타났다. 따라서 10-39세가 40% 미만의 낮은 면역수준을 보이고 이들은 출생코호트 1970년대~1990년대에 해당된다. 결국 이들 코호트가 나이 들면서 우리나라 성인의 면역수준이 더욱 낮아질 것으로 보인다(6,7). 이러한 연령별 항체 수준은 2015년의 국민건강영양조사 결과에서도 20대는 12.6%, 30대는 31.8%, 40대는 80.3%로 나타나 큰 차이가 없었다 (Table 1)(6,8).

(대규모 유행의 원인, causes of outbreak)

A형 간염은 사람 간 밀접접촉에 의한 전파와 수인성 및 식품매개 전파가 주된 전파 경로이다. 지금까지 나타난 한국 A형간염 유행의 전파경로는 사람간 전파와 께 오염된 식수(9, 10), 오염된 조개젓갈에 의한 감염 등이다(11-14) 그런데

예년과 달리 이렇게 유행의 규모가 커지고 있는 것은 무엇 때문일까? 가장 중요한 것은 다음 4가지로 정리해볼 수 있다.

첫째, 성인의 A형간염 집단 면역수준이 낮아지고 있다(15). 한국의 1960년대 출생코호트까지는 어렸을 때 자연 감염으로 인한 면역 획득이 활발했던 세대이다. 그러나 1970년 이후 출생자들은 위생수준의 향상으로 점차 A형간염 면역 수준이 낮아지고 있다. 이는 1990년대 말에 우리나라에 A형 간염 예방접종이 도입되고 2012년 출생자부터는 국가무료예방접종(NIP)을 시행하였기 때문에 1990년대 출생코호트 까지는 면역 수준이 매우 낮은 상황이다. 따라서 시간이 지날수록 면역이 있는 1960년대이전 성인은 줄어들고 면역이 없는 성인들의 비율이 늘어나고 있는 것이다. 2019년 시점에서 보면 가장 왕성한 사회활동을 하는 성인 그룹인 20-49세까지가 면역이 낮은 1970-1990년대 출생코호트인 것이다.

둘째, 한국 식문화의 변화이다. 이번 유행에서 A형간염 바이러스가 확인된 식품은 대부분 중국산 조개젓갈이다(11). 조개젓갈은 한국의 전통식품이지만 최근에는 국내산보다는 중국산이 많이 유통되고 있다. 그런데 조개는 A형 간염 바이러스가 농축될 수 있는 어패류여서 2009년 유행시에도 위험요인으로 제시되었다(16). 젓갈은 익히지 않고 염장을 한 것인데 A형 간염 바이러스는 염장환경에서도 생존이 가능하다(17). 염도가 10%이상은 되어야 A형 간염 바이러스가 불활성 된다는 보고도 있다(18). 또한 아직 A형간염 발생 수준이 높은 중국의 연안지역은 한국보다 오염 가능성이 높은데, 과거 1988년 상하이의 약 30만명에 달하는

Volume: 41, Article ID: e2019038

https://doi.org/10.4178/epih.e2019038

A형간염 유행의 경우에도 익히지 않은 조개를 섭취한 경우가 섭취하지 않은 경우에 비해 22.9배의 높은 발병률을 보였다(19).

셋째, 한국의 공중보건체계가 미약하기 때문이다. 한국의 의료기술은 세계 최고 수준이고 의료 이용률과 의료비 지출은 크게 증가하였으나 상대적으로 공중보건체계는 크게 나아지지 않고 있다. 전세계적으로 신종감염병이 유행하고, 해외유입감염병과 항생제 내성을 포함한 병원관련감염병의 위협이 커지고 있지만 이에 대응하기 위한 공공보건관리체계나 공공보건의료인력, 공공보건에 대한 국가 지출은 미미하다. 이러한 취약성의 결과로 나타난 대표적인 유행이 2015년 메르스 유행이다. 이후 역학조사관 충원과 병원감염 관리를 위한 제도와 정책이 도입되고 있으나 속도는 더디고 보건의료 인력 양성과 의료전달체계, 공중보건조직 체계 공고화 등의 근본적인 문제는 바뀌지 않고 있다. 이번 A형간염 유행은 2009년 대유행을 겪었을 때 이미 예견되었던 바이다. 당시에도 1970-90년대생의 낮은 면역 수준이 원인으로 지목되었고 해당 연령군의 면역을 높일 따라잡기 예방접종시행이 필요하며 가장 비용효과적이라고 제시되었지만(20, 21) 실제 정책은 기존에 선택접종으로 50% 정도의 접종률을 나타내던 1세 접종을 필수예방접종으로 하여 90% 수준으로 높이는 것이었다. 사실 이러한 정책은 모델링 연구에서 가장 비효율적인 정책이었다. 결국 2019년 현재 시점에서 보면 만 7세아동 까지의 면역수준은 크게 높아졌지만 그 이상 연령의 학생들과 성인의 낮은 면역 수준은 계속 방치되었다. 2010년 말 A형간염을 수인성 및 식품매개 감염병인 1군 감염병으로 지정한 것은

역학조사와 체계적 관리의 필요성 때문인데 10년 가까운 시간이 지나는 동안 역학조사와 체계적 관리가 향상되었는지 반성과 평가가 필요하다. 호흡기 감염병의 관리체계와 인프라 구축에 밀려 집단 급식소 등에서 수인성 및 식품매개 감염병 집단발생이 매년 증가하고 있음에도 수인성 및 식품매개 감염병의 역학조사와 관리, 예방은 사실상 방치되고 있는 것은 아닌지 되돌아보아야 한다. 수인성 및 식품매개 감염병에 대한 역학조사와 대응체계를 잘 갖추기 위해서는 광역자치단체와 기초자치단체의 감염병 대응 역량의 실질적 향상이 절실히 요구된다.

넷째, 공중보건정책의 효율적 조정도 매우 부족하다. 예를 들어 우리나라 국방부에서는 군대에서는 반복되는 A형 간염 유행에 대처하기 위해서 2013년부터 신병 대상으로 A형 간염 1회 접종을 시작하여 2015년부터는 모든 신병에 대해서 1회 접종을 시행하고 있다(22). 그 효과로 군대에서 A형 간염 유행은 크게 줄어들었다. 다만 아쉬운 것은 1회 접종만으로는 70% 정도만 면역이 생기므로 2차 접종을 시행해야 하는데 이러한 정보를 연계하여 관리할 시스템이 그동안 없었다. 그러다보니 제대 후 사회에 나와서 A형 간염에 다시 걸릴 가능성이 있다. 또한 이미 예방접종을 2차까지 맞은 신병들도 등록자료가 없거나 자료 연계가 안되어 있어 불필요한 재접종을 받고 있는 것이다. 다행이 이 문제는 올해 질병관리본부와 국방부의 예방접종기록 연계 협약이 이루어져서 조만간 보완이 될 것으로 보인다. 하지만 16세 (백신 종류에 따라 18세까지) 미만 청소년이라면 소아용 백신을 맞아도 되므로 백신 비용을 반으로

줄일 수 있고 청소년 집단면역 수준을 일찌감치 높여 A형 간염 유행을 막는데 기여할 수 있는데도 불구하고(23) 신병에게 성인용 백신 접종을 하는 것은 크나큰 낭비이다.

A형 간염은 소아부터 성인까지 모두 예방접종을 관리해야 하는데 우리나라의 소아 필수예방접종에 대해서는 질병관리본부에서 2012년에 민간의료기관에서도 무료 접종을 시행하면서 접종등록률이 90%이상으로 높아져 비교적 관리가 잘 되고 있다. 하지만 필수예방접종이 아닌 소아 백신(예를 들어 로타바이러스 백신이나 BCG경피용 백신 등)이나 성인 백신 (65세이상 인플루엔자 접종과 폐구균 백신을 제외한 대부분의 백신)에 대해서는 접종 등록 관리가 거의 이루어지지 않고 있다. 그렇다보니 성인이 본인부담으로 A형 간염 예방접종을 받았어도 본인이 기억하지 못하거나 증명할 자료를 가지고 있지 않으면 불필요한 검사나 중복 접종을 하게 되는 경우도 많다. 성인 예방접종 항목이 많아지고 고령자 비율이 증가하고 있어 성인 예방접종등록관리의 중요성이 더욱 커지고 있다.

(A형 간염 유행 관리방법, countermeasures of outbreak)

감염병 유행을 막기위한 기본 정책은 3가지이다. 첫번째는 감염원을 없애는 것이다. 즉, A형 간염에 걸려서 바이러스를 배출하고 있는 사람을 찾아내어 격리하는 것이다. 따라서 A형 간염 환자 조기발견을 통하여 가능한 빨리 치료를 하고 다른 사람들과의 접촉을 줄이도록 하는 것이 필요하다. 하지만 A형 간염의

https://doi.org/10.4178/epih.e2019038

초기 증상은 감기처럼 비특이적이고 일부는 A형 간염에 걸렸어도 증상이 없이 바이러스만 배출하는 경우도 있어서 A형 간염 환자를 모두 찾아내 격리하는 것은 어렵다.

두번째는 감염 전파를 차단하는 것이다. 즉, 바이러스에 오염된 물이나 음식, 화장실 손잡이, 일상 생활 도구 등을 모두 찾아서 제거하거나 소독하여 감수성자가 접촉하지 않도록 하는 것이다. 이를 위해서는 A형 간염 역학조사를 철저히 하여 오염된 음식이나 물을 찾아서 더 이상 유통되거나 사용하지 않도록 하여 추가 환자 발생을 막는 것이 중요하다. 또한 화장실 사용 후, 일상생활에서 항상 손을 깨끗이 씻도록 하는 위생습관 역시 중요하다. 하지만 이 방법만으로 A형 간염 유행을 완벽히 차단하는 것은 불가능하다.

마지막 세번째는 감염가능성이 있는 사람들의 면역수준을 높여서 바이러스가 몸에 들어오더라도 감염되지 않도록 하는 것이다. 그러기 위해서는 어렸을 때 감염되어 자연 면역을 획득하거나 예방접종을 통해서 인공 면역을 획득하면 된다. 우리나라는 이제는 어렸을 때 자연 면역을 획득하기 어려운 시대이고 최근에는 영유아 예방접종을 시행하고 있으므로 문제는 성인들의 면역 수준을 높이기 위한 방법이 필요하다(24). 하지만 모든 사람들에게 A형 간염 예방접종을 시행하는 것은 현실적으로 어렵다. 따라서 A형 간염 유행을 막기위해서는 위 3가지 정책을 모두 사용하여야 한다. 하지만 어느 정책을 중점적으로 시행할 것인지는 국가별 상황에 따라서 달라질 수 있다. 특히 예방접종 정책은 국가별 A형 간염 발생률 수준에 따라서 달라진다. WHO에서는 A형 간염 발생률이 아주

Volume: 41, Article ID: e2019038

https://doi.org/10.4178/epih.e2019038

낮고, 유행도 드물게 일어나는 나라에서는 A형 간염 고위험군 (즉, 유병률 높은 지역 여행자, 혈우병 환자, 남성 동성애자, 불법약물남용자, 영장류와 접촉하는 직업 등)은 예방접종을 해야한다고 권고하고 있다(25). A형 간염 발생률이 높은 나라에서는 어렸을 때 이미 감염되어 면역을 획득하는 사람이 많으므로 대규모 백신접종의 효과가 별로 없어 권고하지 않는다. 그러나 A형 간염 발생률이 높은 수준에서 낮은 수준으로 변하는 단계에 있어서 연령이나 지역별로 면역수준의 차이가 큰 나라에서는 대규모 백신 접종이 비용-효과적이라고 보고있다(25). 한국은 이 단계에 해당되는 나라로 1970-90년대생의 면역수준이 상대적으로 낮고, 특히 도시지역에서 더 낮게 나타나고 있다. 또한 A형 간염은 연령이 증가함에 따라 합병증 발생이 많고 치명률이 증가하므로 향후 유행에서는 A형간염 치명률이 점점 높아질 수 있다. 따라서 대규모 따라잡기 백신 접종 정책 도입이 필요하다. 20-40대 성인을 대상으로 하는 대규모 따라잡기 백신 접종 정책의 도입을 위해서는 예산 확보, 백신 수급, 낮은접종률 등이 걸림돌이 될 수 있으나 5년 이상의 장기 계획을 수립하여 추진한다면 극복 가능할 것이다. 단기적으로는 보건소 등의 기관에서 성인백신을 병의원보다 값싸게 실비 제공하여 비용부담으로 예방접종을 받지 못하는 사람들에게 대안을 마련해주는 것이 필요하다.

결론

이번 한국의 A형 간염 유행은 많은 시사점을 준다. 한국과 같이 빠른

경제성장으로 환경위생 개선이 이루어지는 개발도상국들은 A형 간염 유행이라는 예기치않은 복병을 만나게 될수있다. 너무 늦지않게 영유아 예방접종을 도입하여 특정 연령층의 집단면역이 크게 낮아지지 않도록 하고 예방접종 등록 관리시스템을 도입하여 효율적인 A형 간염 관리를 준비할 필요가 있다.

한국은 성인 집단면역수준이 낮은데 비하여 인구밀도가 높고, 해외 여행자 규모는 해마다 크게 증가하고 있고, 학교나 직장에서의 밀접한 접촉, 높은 의료이용률, 회식문화, 어패류 생식 등 A형 간염 유행의 위험이 다른 나라보다 높다고 할 수 있다. 이러한 특징은 신종감염병, 병원관련감염병 등의 유행 위험도 매우 높음을 의미한다. 그런데 한국의 의료기술 수준과 국민의 보건의료에 대한 높은 기대와는 달리 공공보건의료체계, 공중보건관리체계의 수준은 크게 부족한 것이 현실이다.

감염병이 하나 유행할때마다 그에 대한 땜질식 처방은 비효율적이다. 향후 어떤 감염병이라도 미리 예방하고, 조기발견하여 유행의 확산을 방지하고 효율적인 감염병 관리를 통해 피해를 최소화 하기 위해서는 이제라도 근본적으로 공중보건관리 체계를 선진화 하여야 한다.

참고문헌

1. KCDC. 2009 Communicable Disease Surveillance Yearbook. 2010.
2. KCDC. 2010 Infectious Diseases Surveillance Yearbook. 2011.
3. KCDC. Infectious Disease Portal [Available from: http://www.cdc.go.kr/npt/biz/npp/ist/simple/simplePdStatsMain.do.
4. Martin A, Lemon SM. Hepatitis A virus: from discovery to vaccines. Hepatology. 2006;43(2

Suppl 1):S164-72.
5. Seo JY, Choi S, Choi B, Ki M. Age-period-cohort analysis of hepatitis A incidence rates in Korea from 2002 to 2012. Epidemiol Health. 2016;38:e2016040.
6. Kim KA, Lee A, Ki M, Jeong SH. Nationwide Seropositivity of Hepatitis A in Republic of Korea from 2005 to 2014, before and after the Outbreak Peak in 2009. PLoS One. 2017;12(1):e0170432.
7. Yoon JG, Choi MJ, Yoon JW, Noh JY, Song JY, Cheong HJ, et al. Seroprevalence and disease burden of acute hepatitis A in adult population in South Korea. PLoS One. 2017;12(10):e0186257.
8. Lim J, Kim K, Choi S, Park SM. The effectiveness and limitation of the national childhood hepatitis A vaccination program in the Republic of Korea: Findings from the Korean National Health and Nutrition Examination Survey (KNHANES), 2015. PLoS One. 2017;12(12):e0189210.
9. Ryu S, Won SA, Uh J, Song JY. Hepatitis A Virus Infection from a Contaminated Tap of Ground Water Facility in a Neighborhood Park, Republic of Korea. Infect Chemother. 2019;51(1):62-6.
10. Shin E, Kim JS, Oh KH, Oh SS, Kwon M, Kim S, et al. A waterborne outbreak involving hepatitis A virus genotype IA at a residential facility in the Republic of Korea in 2015. J Clin Virol. 2017;94:63-6.
11. KCDC. 2019 Publication on in-depth epidemiological investigation following Hepatitis A outbreak [press release]. 11 September 2019.
12. PusanCity. Busan Metropolitan City, Publication on Hepatitis A collective outbreak investigation results [press release]. 2 September 2019.
13. KCDC. Hepatitis A virus detection in salted clams from China, KCDC reports. [press release]. 25 June 2019.
14. MFDS. Recall action of imported salted clams with Hepatitis A virus, Korea MFDS reports [press release]. 19 July 2019.
15. Yoon EL, Sinn DH, Lee HW, Kim JH. Current status and strategies for the control of viral hepatitis A in Korea. Clin Mol Hepatol. 2017;23(3):196-204.
16. Seo JY, Choi BY, Ki M, Jang HL, Park HS, Son HJ, et al. Risk factors for acute hepatitis A infection in Korea in 2007 and 2009: a case-control study. J Korean Med Sci. 2013;28(6):908-14.
17. Sánchez G. Processing Strategies to Inactivate Hepatitis A Virus in Food Products: A Critical Review. Comprehensive Reviews in Food Science and Food Safety. 2015;14(6):771-84.
18. Park SY, Ha S-D. Influence of NaCl on the inactivation of murine norovirus-1 and hepatitis A virus in the Korean traditional salted oyster product "Eoriguljeot" during storage. Food Research International. 2014;62:382-7.
19. Halliday ML, Kang LY, Zhou TK, Hu MD, Pan QC, Fu TY, et al. An epidemic of hepatitis A

attributable to the ingestion of raw clams in Shanghai, China. J Infect Dis. 1991;164(5):852-9.

20. Kim K, Jeong BG, Ki M, Park M, Park JK, Choi BY, et al. The costs of hepatitis A infections in South Korea. Epidemiol Health. 2014;36:e2014011.

21. Ki M, Hepatitis_research_team. Economic Analysis of Vaccination and Development of Guidelines for Control of Hepatitis A Virus Infection, and Epidemiologic Analysis and Development of Control Strategy of Hepatitis C Virus Infection (This work was supported by the KCDC). Report No2008-E00426-00. 2009.

22. Heo JY, Choe KW, Yoon CG, Jeong HW, Kim WJ, Cheong HJ. Vaccination policy in Korean armed forces: current status and future challenge. J Korean Med Sci. 2015;30(4):353-9.

23. Hankin-Wei A, Rein DB, Hernandez-Romieu A, Kennedy MJ, Bulkow L, Rosenberg E, et al. Cost-effectiveness analysis of catch-up hepatitis A vaccination among unvaccinated/partially-vaccinated children. Vaccine. 2016;34(35):4243-9.

24. Chironna M, Prato R, Sallustio A, Martinelli D, Tafuri S, Quarto M, et al. Hepatitis A in Puglia (South Italy) after 10 years of universal vaccination: need for strict monitoring and catch-up vaccination. BMC infectious diseases. 2012;12:271.

25. WHO position paper on hepatitis A vaccines: June 2012-recommendations. Vaccine. 2013;31(2):285-6.
